# Supplementary material for: Early Detection of Macular Atrophy Automated Through 2D and 3D Unet Deep Learning
Source: Bioengineering (Basel). 2024 Nov 25;11(12):1191. doi: 10.3390/bioengineering11121191 (PMC11726850; doi:10.3390/bioengineering11121191)
Supplement: Supplementary file 1 [file bioengineering-11-01191-s001.zip › bioengineering-3292157-supplementary.pdf]

Learning curve analysis of 2D model

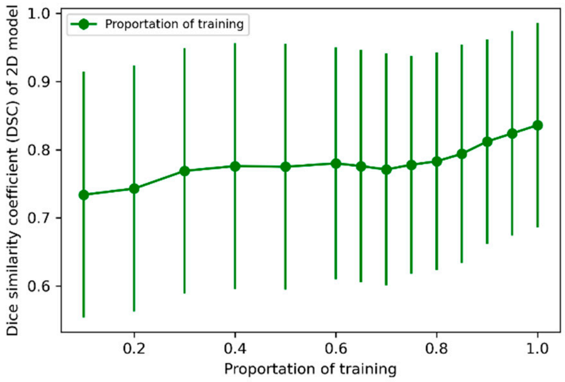

Figure S1. The DSC score showed a gradual upward trend with increasing training datasets, but the sample size was still not enough eventually. Therefore, data augmentation was used to improve the performance of models.

Performance of 3D models during each round of cross-validation

**Multiclass (cross validation)**	**One-dimensional evaluation**			
	DSC	Precision	Recall	F1 score
2D_Unet	0.87±0.15	0.86±0.17	0.90±0.16	0.87±0.15
3D_Unet_Round 1	0.90±0.11	0.89±0.15	0.91±0.17	0.90±0.15
3D_Unet_Round 2	0.90±0.13	0.89±0.13	0.89±0.15	0.89±0.13
3D_Unet_Round 3	0.89±0.13	0.87±0.15	0.91±0.16	0.89±0.15
3D_Unet_Round 4	0.89±0.18	0.88±0.11	0.90±0.12	0.89±0.12
3D_Unet_Round 5	0.90±0.13	0.89±0.14	0.91±0.13	0.90±0.13

Table S1. Comparison of the performance of multiclass model between 2D and 3D models

Absence of EZ (cross validation)	One-dimensional evaluation			
	DSC	Precision	Recall	F1 score
2D_Unet	0.94±0.10	0.94±0.11	0.95±0.10	0.94±0.10
3D_Unet_Round 1	0.96±0.03	0.95±0.05	0.96±0.05	0.96±0.03
3D_Unet_Round 2	0.95±0.04	0.93±0.07	0.98±0.03	0.95±0.04
3D_Unet_Round 3	0.95±0.03	0.95±0.05	0.96±0.05	0.95±0.03
3D_Unet_Round 4	0.94±0.04	0.91±0.08	0.98±0.03	0.94±0.04
3D_Unet_Round 5	0.95±0.04	0.94±0.06	0.97±0.04	0.95±0.04

Table S2. Comparison of the performance of absence of EZ between 2D and 3D models

Absence of RPE (cross validation)	One-dimensional evaluation			
	DSC	Precision	Recall	F1 score
2D_Unet	0.90±0.12	0.92±0.13	0.92±0.14	0.91±0.12
3D_Unet_Round 1	0.95±0.08	0.93±0.12	0.99±0.04	0.96±0.08
3D_Unet_Round 2	0.96±0.07	0.95±0.10	0.97±0.06	0.96+0.07
3D_Unet_Round 3	0.96±0.06	0.96±0.08	0.96±0.07	0.96±0.06
3D_Unet_Round 4	0.94±0.07	0.94±0.12	0.96±0.06	0.94±0.07
3D_Unet_Round 5	0.93±0.10	0.89±0.16	0.99±0.02	0.93±0.10

Table S3. Comparison of the performance of absence of RPE between 2D and 3D models

**Hypertransmission >=250um (cross validation)**	**One-dimensional evaluation**			
	DSC	Precision	Recall	F1 score
2D_Unet	0.88±0.16	0.87±0.20	0.93±0.13	0.88±0.16
3D_Unet_Round 1	0.92±0.11	0.98±0.03	0.88±0.16	0.92±0.10
3D_Unet_Round 2	0.96±0.07	0.94±0.11	0.95±0.10	0.95±0.07
3D_Unet_Round 3	0.94±0.08	0.98±0.05	0.92±0.12	0.94±0.08
3D_Unet_Round 4	0.96±0.06	0.96±0.07	0.96±0.07	0.96±0.06
3D_Unet_Round 5	0.92±0.09	0.98±0.05	0.89±0.14	0.92±0.09

Table S4. Comparison of the performance of hypertransmisstion>=250um between 2D and 3D models

**Hypertransmission <250um (cross validation)**	**One-dimensional evaluation**			
	DSC	Precision	Recall	F1 score
2D_Unet	0.63±0.20	0.65±0.26	0.71±0.24	0.63±0.20
3D_Unet_Round 1	0.78±0.15	0.77±0.25	0.79±0.18	0.77±0.15
3D_Unet_Round 2	0.77±0.28	0.74±0.25	0.78±0.21	0.76±0.28
3D_Unet_Round 3	0.78±0.21	0.77±0.18	0.76±0.16	0.77±0.21
3D_Unet_Round 4	0.78±0.18	0.78±0.29	0.78±0.23	0.78±0.18
3D_Unet_Round 5	0.78±0.18	0.75±0.28	0.78±0.22	0.76±0.18

Table S5. Comparison of the performance of hypertransmisstion<250um between 2D and 3D models

**Interruption of EZ (cross validation)**	**One-dimensional evaluation**			
	DSC	Precision	Recall	F1 score
2D_Unet	0.65±0.20	0.65±0.25	0.72±0.24	0.65±0.20

3D_Unet_Round 1	0.76±0.20	0.77±0.26	0.76±0.20	0.77±0.20
3D_Unet_Round 2	0.76±0.24	0.73±0.24	0.79±0.14	0.76±0.17
3D_Unet_Round 3	0.77±0.22	0.76±0.24	0.77±0.20	0.76±0.21
3D_Unet_Round 4	0.77±0.22	0.77±0.21	0.78±0.20	0.77±0.20
3D_Unet_Round 5	0.77±0.24	0.76±0.24	0.78±0.21	0.77±0.20

Table S6. Comparison of the performance of interruption of EZ between 2D and 3D models

**Interruption of RPE (cross validation)**	**One-dimensional evaluation**			
	DSC	Precision	Recall	F1 score
2D_Unet	0.77±0.20	0.78±0.22	0.82±0.22	0.77±0.20
3D_Unet_Round 1	0.81±0.20	0.81±0.19	0.83±0.21	0.82±0.21
3D_Unet_Round 2	0.81+0.21	0.79±0.17	0.82±0.21	0.80±0.20
3D_Unet_Round 3	0.79±0.19	0.80±0.19	0.82±0.23	0.81±0.20
3D_Unet_Round 4	0.80±0.20	0.81±0.20	0.82±0.21	0.81±0.20
3D_Unet_Round 5	0.79±0.19	0.81±0.20	0.82±0.20	0.81±0.19

Table S7. Comparison of the performance of interruption of RPE between 2D and 3D models
